# Supplementary material for: Leukemia relapse via genetic immune escape after allogeneic hematopoietic cell transplantation
Source: Nat Commun. 2023 May 31;14:3153. doi: 10.1038/s41467-023-38113-4 (PMC10232425; doi:10.1038/s41467-023-38113-4)
Supplement: Supplementary file 1 — Supplementary Information [file 41467_2023_38113_MOESM1_ESM.pdf]

Supplementary Information

Leukemia relapse via genetic immune escape after allogeneic hematopoietic cell transplantation

Simona Pagliuca,[1,2,3]* Carmelo Gurnari,[1,4]* Colin Hercus,[5] Sébastien Hergalant,[6] Sanghee Hong,[7] Adele Dhuyser,[8,3] Maud D'Aveni,[2,3] Alice Aarnink,[8,3] Marie Thérèse Rubio,[2,3] Pierre Feugier,[2] Francesca Ferraro,[9] Hetty E. Carraway,[10] Ronald Sobecks,[11] Betty K. Hamilton,[11] Navneet S. Majhail,[12] Valeria Visconte[1] and Jaroslaw P. Maciejewski[1]

[1] Department of Translational Hematology and Oncology Research, Taussig Cancer Institute, Cleveland Clinic, Cleveland, OH
[2]Department of Clinical Hematology, CHRU Nancy, Nancy, France
[3]CNRS UMR 7365, IMoPA, Biopole of University of Lorraine, Nancy, France
[4]Department of Biomedicine and Prevention, PhD in Immunology, Molecular Medicine and Applied Biotechnology, University of Rome Tor Vergata, Rome, Italy
[5]Novocraft Technologies Sdn Bhd, Kuala Lumpur, Malaysia
[6]Inserm UMR_S1256 Nutrition-Genetics-Environmental Risk Exposure, University of Lorraine, 54500 Nancy, France
[7]Division of Hematologic Malignancies and Cellular Therapy, Department of Medicine, Duke University School of Medicine, Durham, NC, USA
[8]Histocompatibility department, CHRU Nancy, Nancy, France
[9]Division of Oncology, Department of Medicine, Washington University School of Medicine in St. Louis, St. Louis, MO, USA
[10]Leukemia program, Hematology Department, Taussig Cancer Institute, Cleveland Clinic, Cleveland, OH, USA
[11]Blood and Marrow Transplant Program, Taussig Cancer Institute, Cleveland Clinic, Cleveland, OH, USA
[12]Sarah Cannon Transplant and Cellular Therapy Network, Nashville, TN, USA

*These authors equally contributed to this work

Table of content

Supplementary methods 4

Study population and data collection 4

Transplant details and prophylaxis 4

DNA isolation 5

HLA mutational analysis 5

Whole exome sequencing and analysis 6

Supplemental figures 8

Supplementary Figure 1: Distribution of HED values in healthy controls. 9

Supplementary Figure 2: Univariable Kaplan-Meier estimates of probability of survival according to class-related heterozygosity and HED categorized scores in matched setting. 10

Supplementary Figure 3: Impact of HED values on relapse in haploidentical hematopoietic cell transplantation (HCT) population. 11

Supplementary Figure 4: Relationship between HED and quantitative characteristics of known TCR specificities. 12

Supplementary Figure 5: Clonotype tracking. 13

Supplementary Figure 6: HED value distribution in HLA mutated and wild type group. 14

Supplementary Figure 7: Batch correction and normalization of bulky RNAseq samples. 15

Supplementary Figure 8: Biological pathways enriched in the downregulated gene groups derived from the differential analysis of post-HCT relapse vs diagnosis 16

Supplementary Figure 9: Single cell analysis of diagnosis and post-transplant relapsed leukemia 18

Supplementary Figure 10: Impact of KIR ligand status on HLA-C locus on post-transplant outcomes. 20

Supplementary Figure 11: Allelic loss events in pre and post-HCT specimens. 21

REFERENCES 22

All the supplementary tables are provided as separate Excel files.

Supplementary Data:

Supplementary Data 1: HLA genotypes and HED computation in healthy controls

Supplementary Data 2: Clinical details of patients sequenced for TCR

Supplementary Data 3: Genes investigated from targeted panels for myeloid associated architecture

Supplementary Data 4: Disease characteristics of patients included in RNAseq study

Supplementary Data 5: Immune gene sets studied through whole exome sequencing

Supplementary Data 6: Non-HLA immune related genes impacted by somatic hits

Supplementary Data 7: KIR ligand status and KIR genotype for selected MRD transplants

Supplementary Data 8: Post-HCT TCRvBeta sequencing

Data Source

Data Source 1: Patient level data

Data Source 2: HLA mutations and losses in patients transplanted for AML/MDS

Data Source 3: HLA transcriptomic dysregulation in paired diagnosis-post-transplant samples

Supplementary methods

Study population and data collection

The review of medical records was approved by institutional ethical committees in agreement with the Helsinki Declaration of 1975, revised in 2008.[1] All patients had been regularly followed from until December 2021 (or death). Pertinent clinical data including age, gender, disease diagnosis and risk, comorbidities, type of transplant, human leukocyte antigen (HLA), conditioning regimen, GVHD prophylaxis, acute and chronic GVHD, infections and other clinical complications were collected. Objective medical data including ancillary testing, laboratory results and medical complications, and medication profiles were abstracted through standardized chart review after each visit.

European leukemia net (ELN) 2017, Revised International Prognostic Scoring System (IPSS-R) and Dynamic International Prognostic Scoring System (DIPSS) were used for the classification of respectively acute myeloid leukemias (AML), myelodysplastic syndrome (MDS) and myelofibrosis. Diseases at diagnosis were classified according to a unified scores including the following categories high (ELN adverse, IPSS-R high and very high and DIPSS high), intermediate (ELN intermediate, IPSS-R intermediate 1 and 2 and DIPSS intermediate 1 and 2) and low (the remaining ELN, IPSS-R and DIPSS categories).

Transplant details and prophylaxis

High-resolution, sequence-specific oligonucleotide primed PCR (PCR-SSO) based, 5 loci HLA-typing (for loci HLA-A, B, C, DRB1, DQB1), was used to select donors for allogeneic HCT (according to the 10-alleles donor/recipient status). Donor types included matched related donor (MRD), matched (MUD) and mismatched unrelated donor (MMUD), and haploidentical (HAPLO) donors. Stem cell sources were peripheral blood (PB) and bone marrow (BM). Myeloablative conditioning regimens (MAC) included busulfan (3.2 mg/kg/day for 4 days) combined with cyclophosphamide (60 mg/kg/day for 2 days)[2] or with fludarabine (30 mg/m2/day for 5 days),[3] or total body irradiation (TBI) of 1200 cGy combined with cyclophosphamide (60 mg/kg/day for 2 days), or busulfan (3.2 mg/kg/day for 2-3 days)

combined with thiotepa (5 mg/kg/day for 2 days) and fludarabine (40 mg/m2/day for 4 days).[4] Reduced-intensity regimens (RIC) included fludarabine-based protocols, according to the disease and age of the recipient. Standard protocols of immunosuppression including cyclosporine (CSA) or tacrolimus (FK) and short-term methotrexate (MTX) or CSA/FK with mycophenolic mofetil (MMF) were used for GvHD prophylaxis. In addition, recipients of unrelated donor transplants received rabbit anti-thymocyte globulin (Thymoglobuline 2.5 mg/kg/day for 2-4 days before transplantation) or anti-lymphocyte globulin (Grafalon 10 mg/kg/day for 1-3 days) according to disease and donor HLA matching.

Severity of acute GvHD was graded according to Glucksberg's criteria[5] whereas the assessment of all patients developing chronic GVHD was made according to the National Institute of Health (NIH) consensus criteria.[6,7]

DNA isolation

Genomic DNA was isolated directly from cryopreserved unfractionated peripheral or bone marrow blood mononuclear cells with the Nuclei Lysis Solution (Promega) according to manufacturer's instructions.

HLA mutational analysis

Details of the bioinformatic approach to investigate somatic HLA mutational status have been described elsewhere.[8] In brief, after obtaining a confident full 4th field typing through NovoHLA typing algorithm (Novocraft Technologies), paired-end reads from either targeting sequencing were directly aligned on a per-patient HLA reference using Novoalign (Novocraft Technologies Sdn Bhd).

This process is performed by allowing i) multi-alignment of high quality reads ii) intermediate options of clipping iii) reasonable mismatch penalties and gap openings facilitating the alignment of reads with a mismatched base or a deletion/insertion. After classical sorting, marking duplicates and indexing procedures, according to Genome Analysis Toolkit V.4 best practices,[9] variant calling was performed using Varscan v2.4 in tumor-only mode.[10] A minimum coverage of 30 reads (>10 reads for the variant allele) was used as a threshold for the detection in the targeted platform while a minimum coverage of 10 reads (>4 for the variant allele) was used for WES samples. This generated a list of variants with false positive

events being still potentially present among true calls. To avoid this background noise, we developed a java tool built on the multi-alignment files provided by the HLA-IPD/IMGT database (version 3.41),[11] to retain only non-polymorphic calls (see supplementary appendix). Average coverage for HLA targeted panel was 834x. VAF threshold for HLA variant calling was 2%. We estimated that under this threshold no reliable variant could be identified in hyper-polymorphic regions such as HLA loci.

Allelic loss was imputed computing the number of reads covering each called heterozygous allele within a given locus. The following formula is used:

$$Log2\frac{Ci}{(\Sigma Ci, Cz)/2}$$

With Ci and Cz representing the read coverage for each allele belonging to the same locus. For structurally similar alleles, we included an adjustment taking into account sequence variation defined as “Variant coverage”, directly computed by the NovoHLA pipeline.

All mean Log2 ratios <-1.5 were retained as confident allelic losses, based on a previous internal validation study on 234 healthy controls and cell lines that did not showed altered regions.[12]

It is noteworthy to clarify that since this algorithm is based on the coverage information for each reference base of each allele, differently from SNP-array platforms, does not allow a proper estimation of the genomic boundaries for 6p loss events but can only identify heterozygous allelic losses. A visualization of this process is reported in **Supplementary Figure 11.**

Whole exome sequencing and analysis

After quality control checks sequencing libraries were generated using Agilent SureSelect Human All Exon Kit (Agilent Technologies, Santa Clara, CA, USA) following manufacturer’s instructions. After capture and enrichment with index tags, products were purified and quantified using AMPure XP system (Beckman Coulter, Beverly, MA, USA) and the Agilent high sensitivity DNA assay on the Agilent Bioanalyzer 2100 system. The prepped libraries were hybridized in the buffer with biotin-labeled probes, and magnetic beads with streptavidin were used to capture the exons of genes. Subsequently, non-hybridized fragments were washed out and probes were digested. The captured libraries were enriched by PCR amplification.

The library was checked with Qubit and real-time PCR for quantification and bioanalyzer for size distribution detection. Quantified libraries will be pooled and sequenced on Illumina platforms with PE150 strategy, according to effective library concentration and data amount required.

After discarding low quality reads (i.e. containing sequencing artifacts, including adapter-related sequences or harboring >10% of uncertain bases) and checking for GC content, Burrows-Wheeler Aligner (BWA, v0.7.17)[13] was utilized to map the paired-end clean reads to the human reference genome (GRCh37), SAMtool v1.8[14] and Picard v2.18.9 (http://sourceforge.net/projects/picard/) were applied for post-alignment processing included sorting, marking of duplicates and indexing according to Genome Analysis Tool Kit (GATK) v.4 best practices. After variant calling detecting single nucleotide variants (SNP) and small insertions and deletions (InDels) through the GATK workflow, variant annotation was performed with ANNOVAR, using the following main databases: RefSeq, Gencode, 1000 Human Genome, Exome Aggregation Consortium (ExAC), Genome Aggregation Database (gnomAD) and exome sequencing project (ESP), dbSNP, COSMIC, OMIM, GWAS.

The resultant annotated variant file, after exclusion of possible germline calls, was queried for the presence of aberrations in a specific gene set including almost 400 immune genes, involved in antigen presenting machinery and immune control (**Table S6**). Only pathogenic or likely pathogenic mutations are retained basing on a scoring system developed using more than 10 prediction tools according to Varsome classifier (https://varsome.com/).[15]

Supplemental figures

Supplementary Figure 1

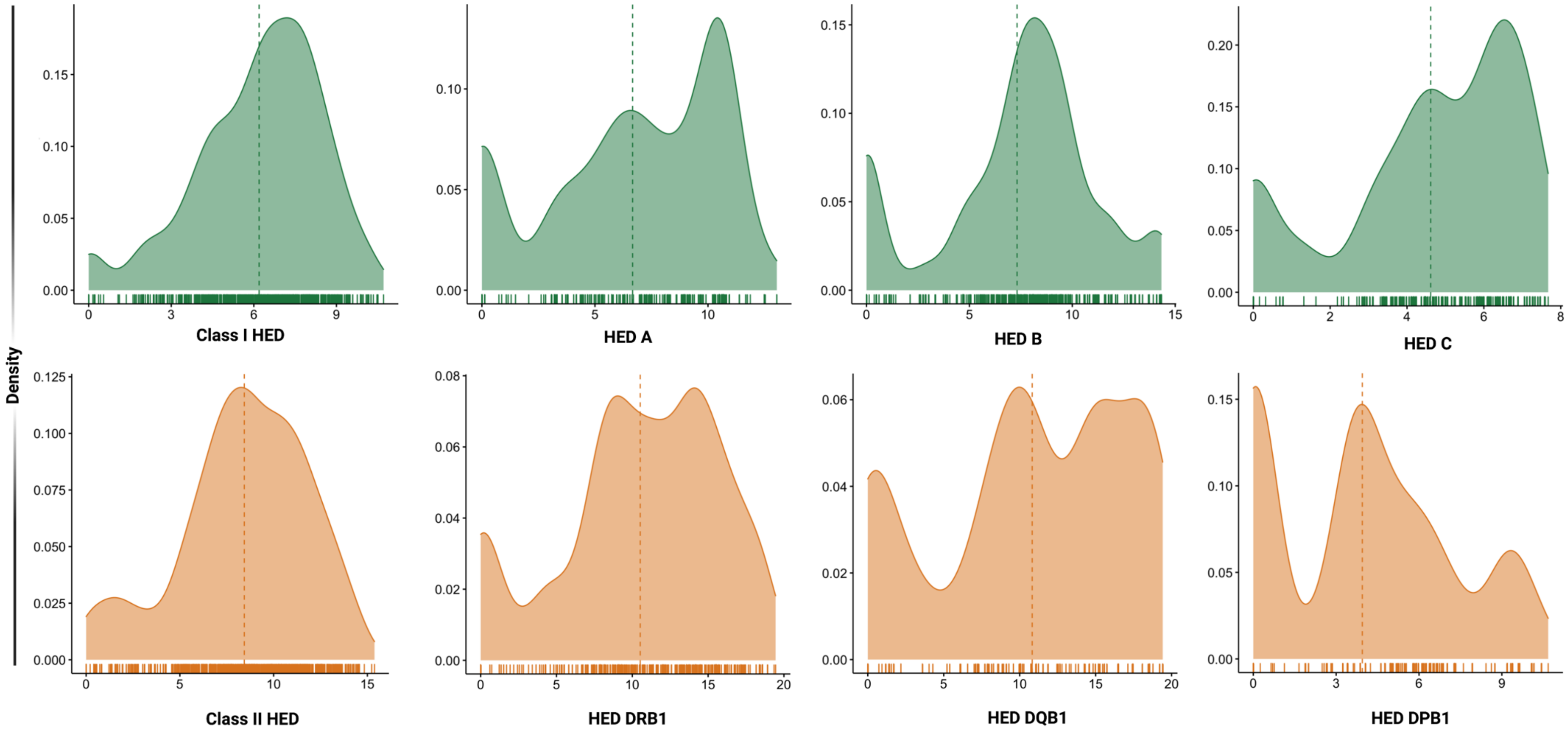

Supplementary Figure 1: Distribution of HED values in healthy controls. For each plot the y-axis represents the density and the x-axis represents each HED score. The frequency of each value (one value per subject in each plot) is shown. The dotted line indicates the median value used as cutoff for the study in our patient population

Supplementary Figure 2

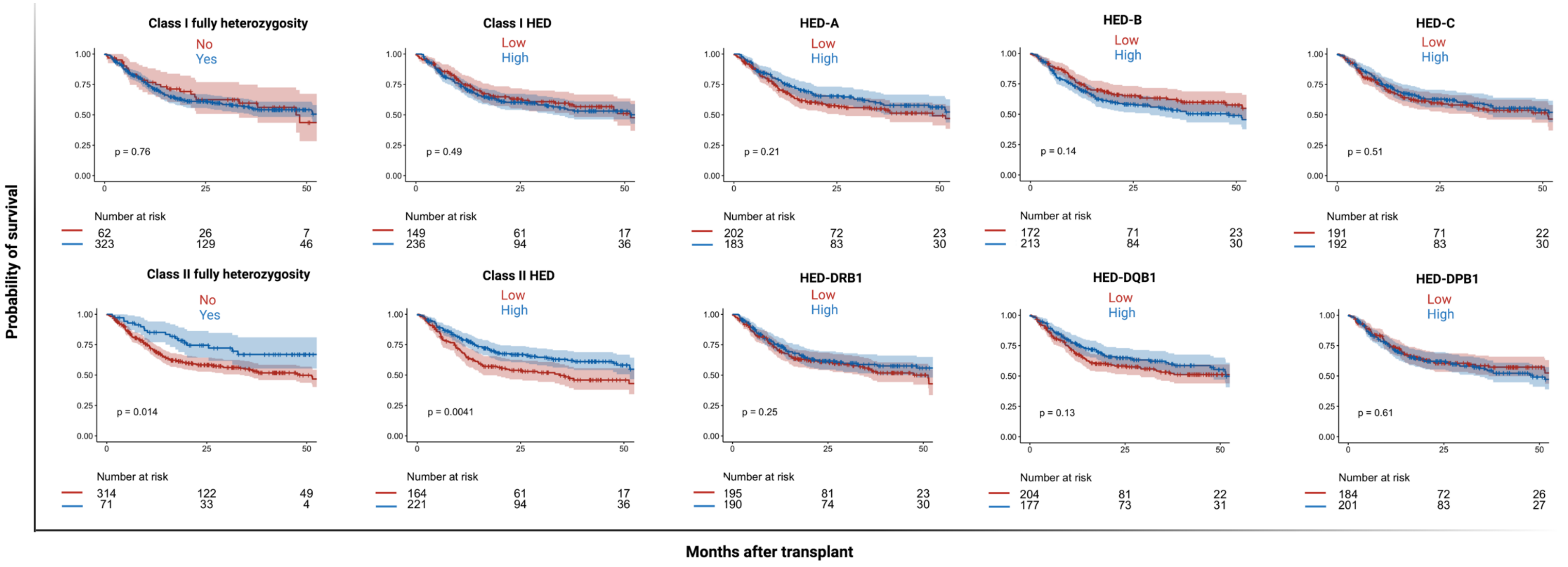

Supplementary Figure 2: Univariable Kaplan-Meier estimates of probability of survival according to class-related heterozygosity and HED categorized scores in matched setting. HED values for each locus are categorized according to the 50th percentile of the corresponding locus specific score in healthy controls (see text). Shaded bands represent 95% confident interval.

Supplementary Figure 3

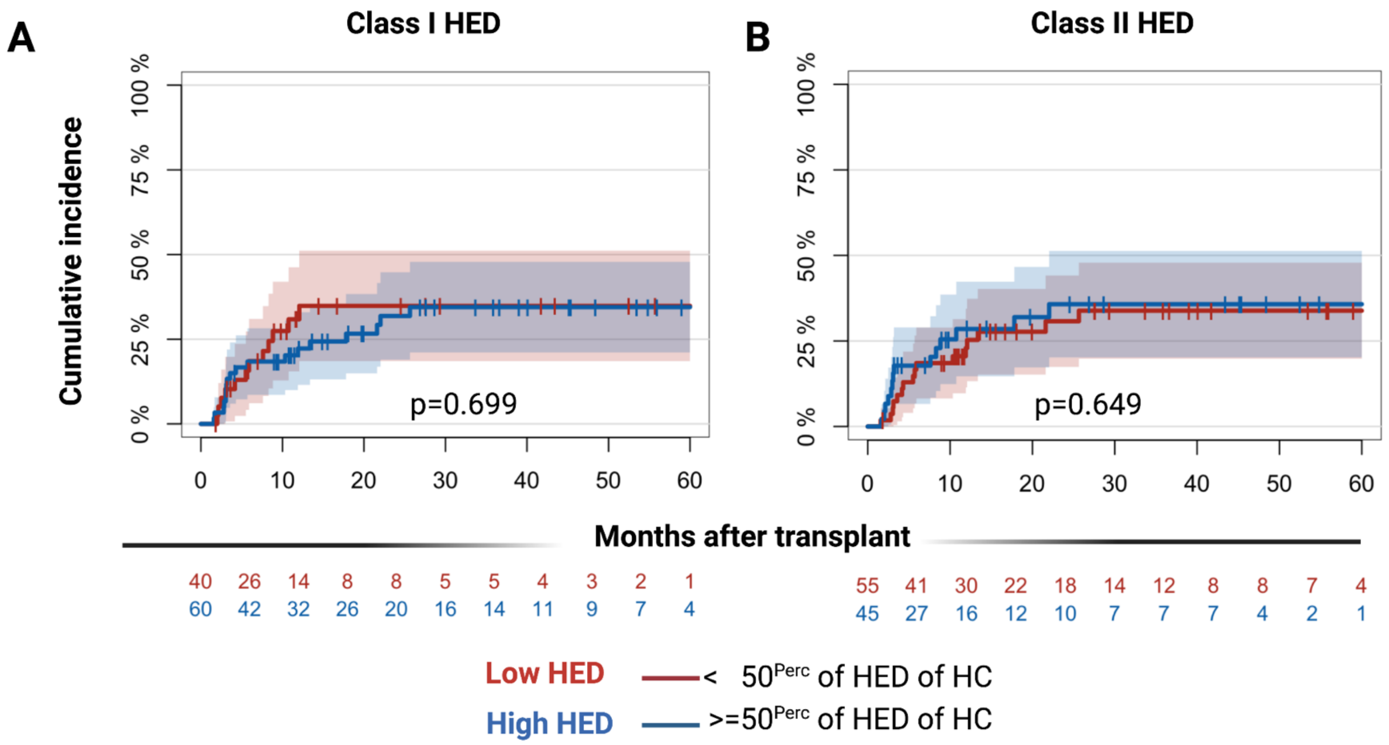

Supplementary Figure 3: Impact of HED values on relapse in haploidentical hematopoietic cell transplantation (HCT) population. Univariable impact of class I (A) and II (B) mean HED categories on the cumulative incidence of relapse in patients receiving a haploidentical HCT for myeloid disorders (N=100). Low and high HED were categorized according to the 50th percentile value of HED scores in healthy controls (see Supplementary Figure 2). Shaded bands represent 95% confident interval.

Supplementary Figure 4

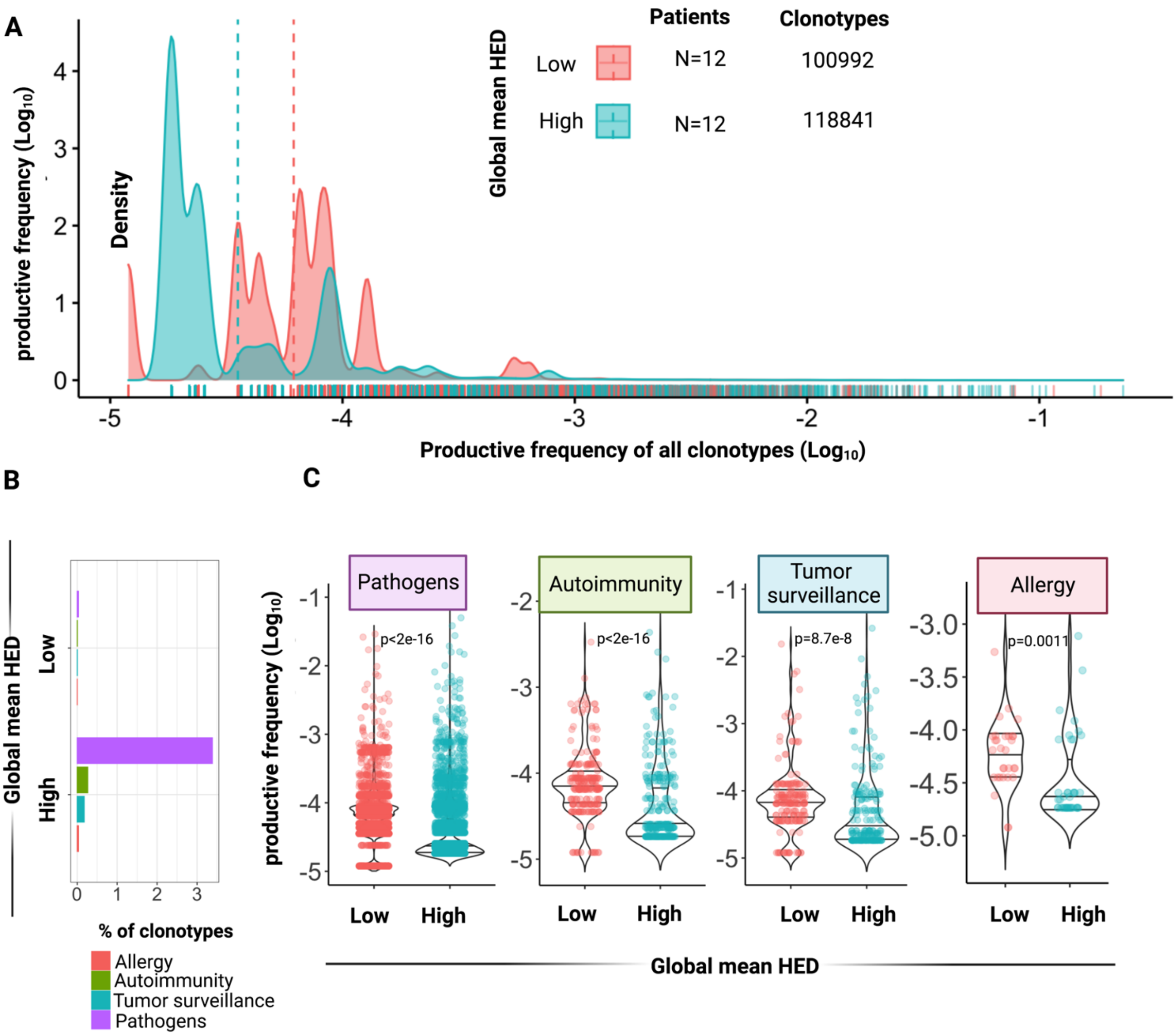

Supplementary Figure 4: Relationship between HED and quantitative characteristics of known TCR specificities.

A) Density plots showing the distribution of the productive frequency (clonal expansion) of post-transplant clonotypes according to HED categories (low: red; high: light blue). 12 patients were analyzed in each group. The number of clonotypes in each dataset (low and high HED) is reported on the top of the figure.

B) Barplot showing the proportion of clonotypes with known specificity in low vs high HED categories (% indicated the fraction of the entire repertoire).

C) Violin plots denoting the distribution of the productive frequency (clonal expansion) of clonotypes with known specificity in low vs high HED groups.
All the p-values were two-sided (wilcoxon signed rank test).

Supplementary Figure 5

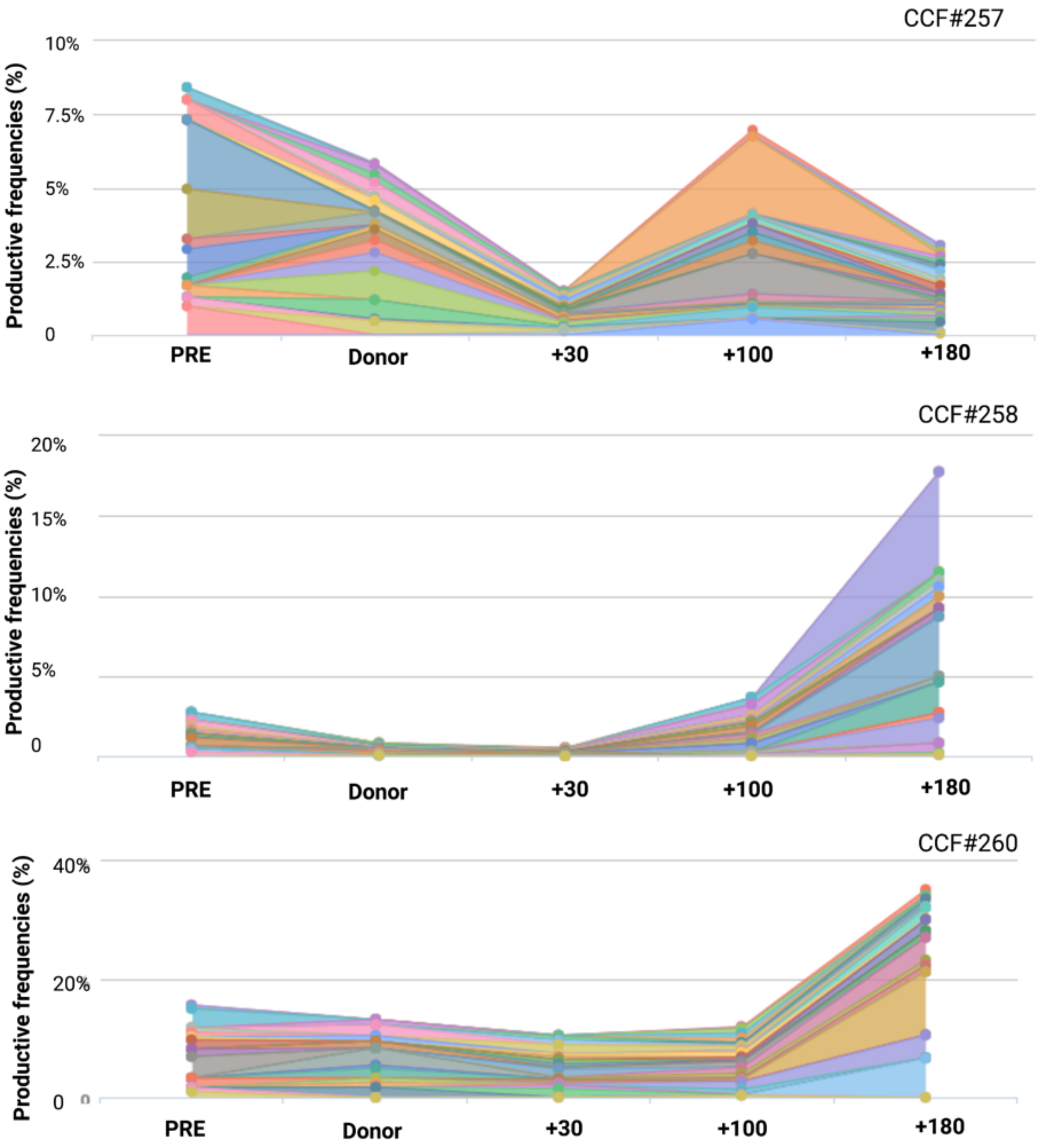

Supplementary Figure 5: Clonotype tracking. Area chart showing the dynamic expansion and contraction of the top ten clonotypes in three patients sequenced longitudinally. Each dot represents a shared expanded clonotype. The area represents the trajectory of the expansion. Groups on X axes represent the timing of the sample collection (5 for each of these patients).

Supplementary Figure 6

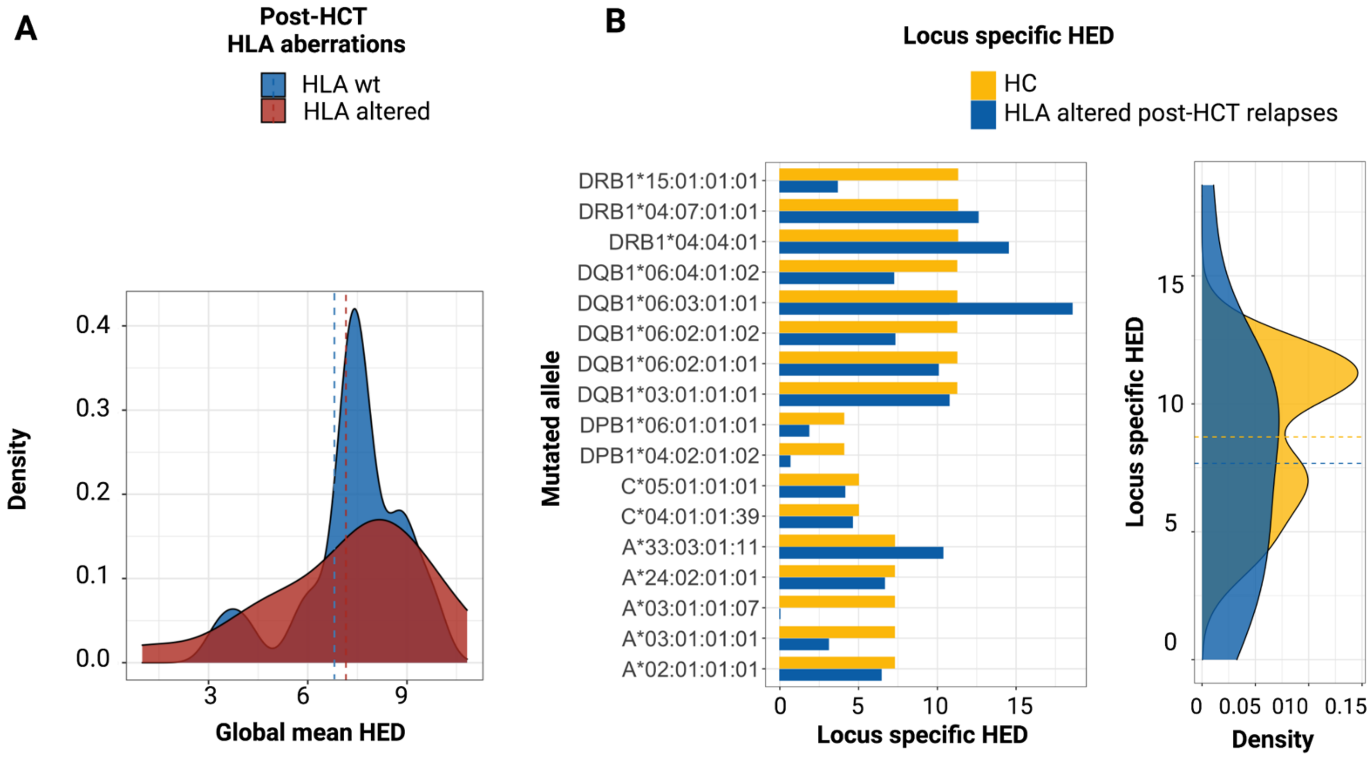

Supplementary Figure 6: HED value distribution in HLA mutated and wild type group.

A) Density distribution (y-axis) of global mean HED values (x-axis) in HLA altered (red) and HLA wild type (wt, blue) group. Median of each group is indicated by the dotted lines.

B) On the left: Locus specific HED for each mutated allele (blue). For comparisons mean values of locus specific HED is captured by the yellow bars. On the right: density distribution of locus specific HED in aberrant loci in patients (blue), vs related loci in healthy controls (yellow).

Supplementary Figure 7

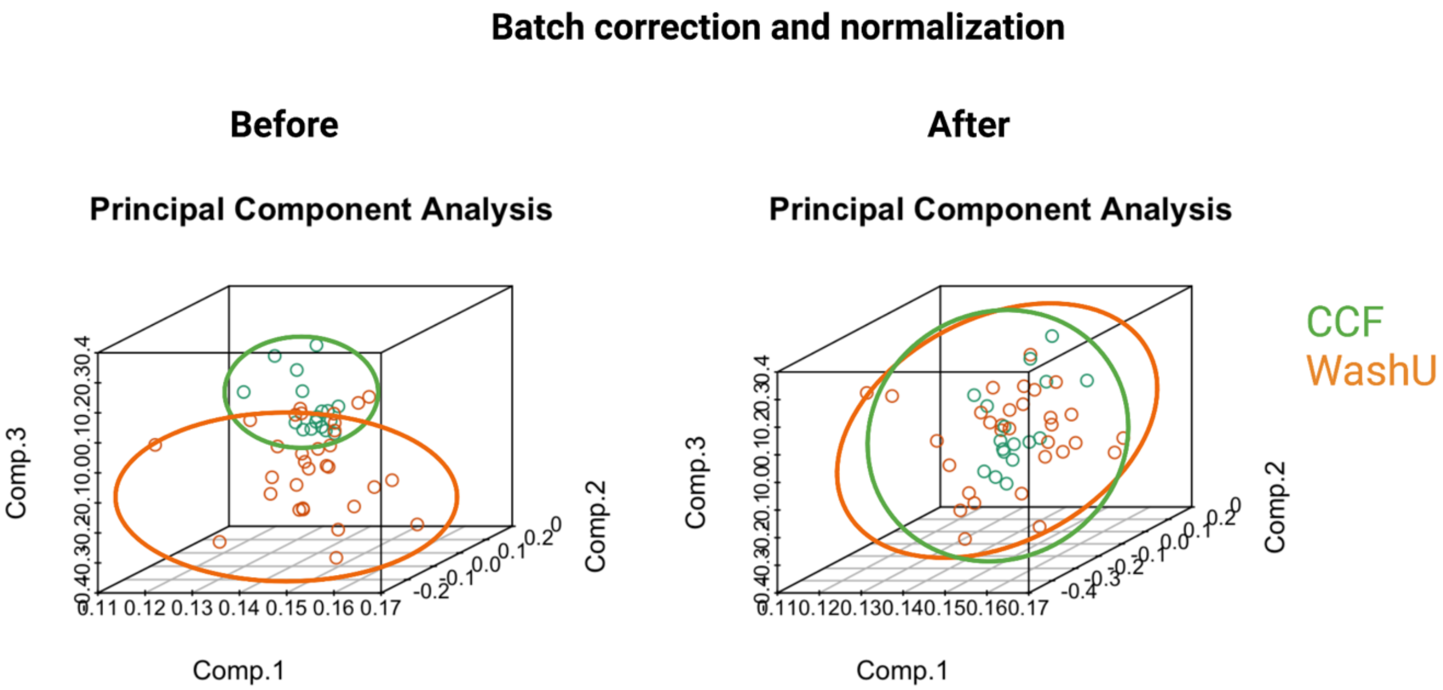

Supplementary Figure 7: Batch correction and normalization of bulky RNAseq samples. 3D principal component plots before (left) and after (right) batch correction and normalization.

Supplementary Figure 8

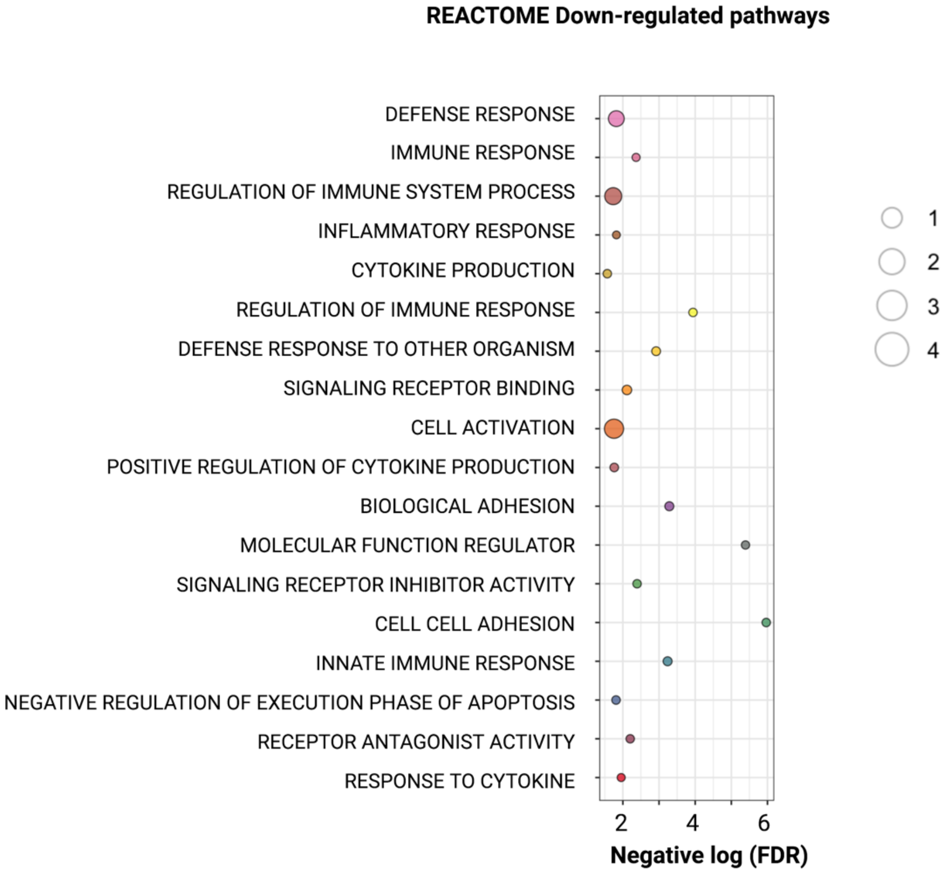

Supplementary Figure 8: Biological pathways enriched in the downregulated gene groups derived from the differential analysis of post-HCT relapse vs diagnosis (N=13 patients, n=26 samples). Gene set enrichment analysis (GSEA) performed with the tool GSEA (Broad Institute, UC San Diego, http://www.gsea-msigdb.org/gsea/index.jsp). X-axis representing the negative logarithm base 10 of the false discovery rate (FDR or q-value, calculated according the Benjamini-Hochberg correction); the y-axis reports the REACTOME pathways enriched in the downregulated gene group. The size of the bubbles depicts the ratio between the number of the genes in the downregulated signature and the number of the genes in the reference gene-set (k/K ratio).

Supplementary Figure 9

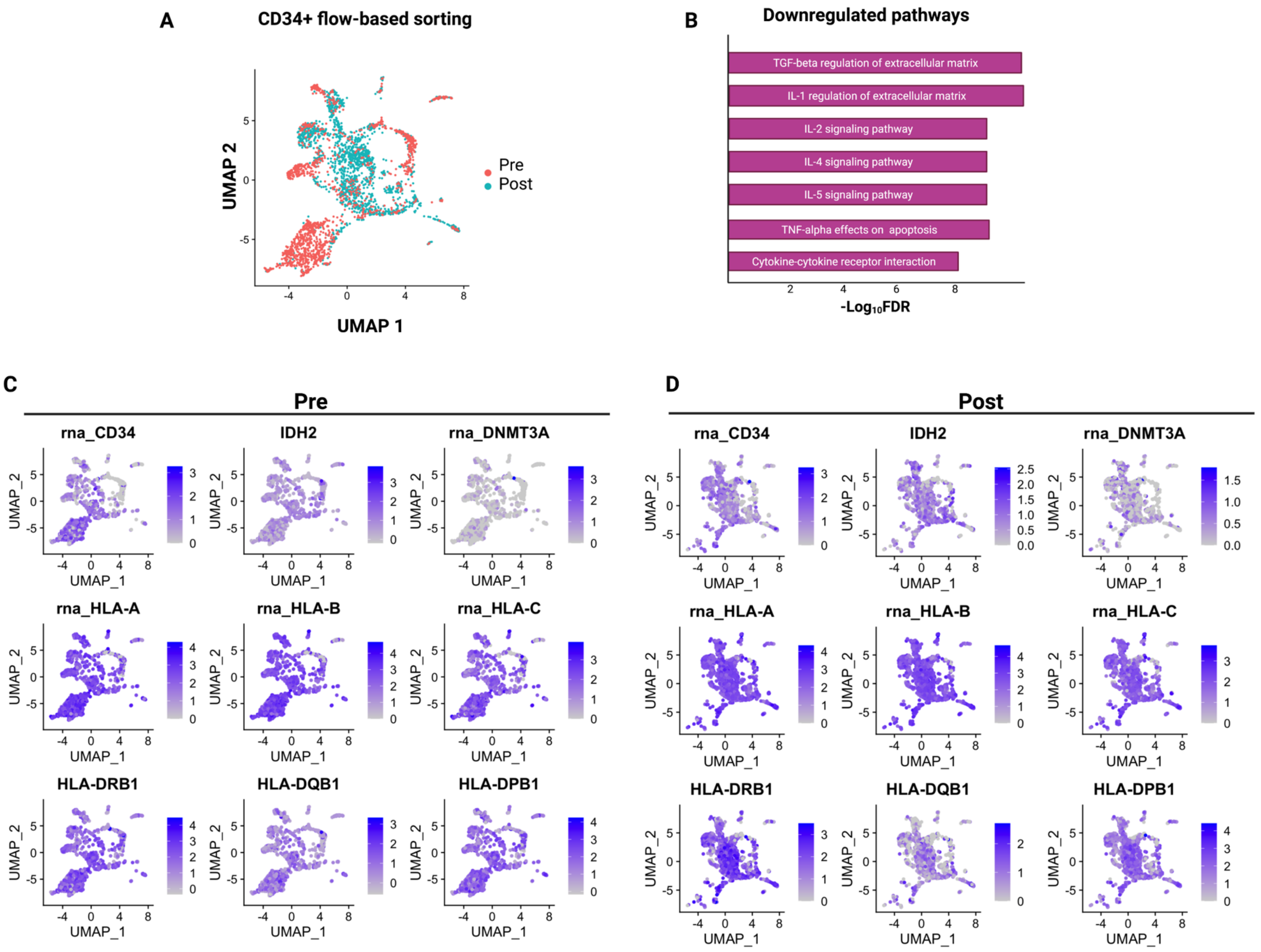

Supplementary Figure 9: Single cell analysis of diagnosis and post-transplant relapsed leukemia

A) Uniform Manifold Approximation and Projection (UMAP) of pre (red dots) and post-transplant (light blue dots) samples from patient #CCF8. Each dot represents a cell. The position of each dot in the graph depends on the intrinsic characteristics of each cell as computed by UMAP algorithm in the purpose of data dimension reduction.
B) Barplots expressing the results of the gene set enrichment analysis (GSEA) resulting from the comparisons between pre and post-transplant samples. X axis indicates the logarithmic false discovery rate (FDR) of the enrichead downregulated pathways. The top 7 pathways are expressed based on the significativity rate.This analysis has been performed with the package EnrichR and the NCATS BioPlanet platform (http://tripod.nih.gov/bioplanet/).
C) Spatial distribution, based on the UMAP projections, of HLA the expression of genes. CD34, IDH2 and DNMT3A are also expressed as proxy for demarking blastic cells.

Supplementary Figure 10

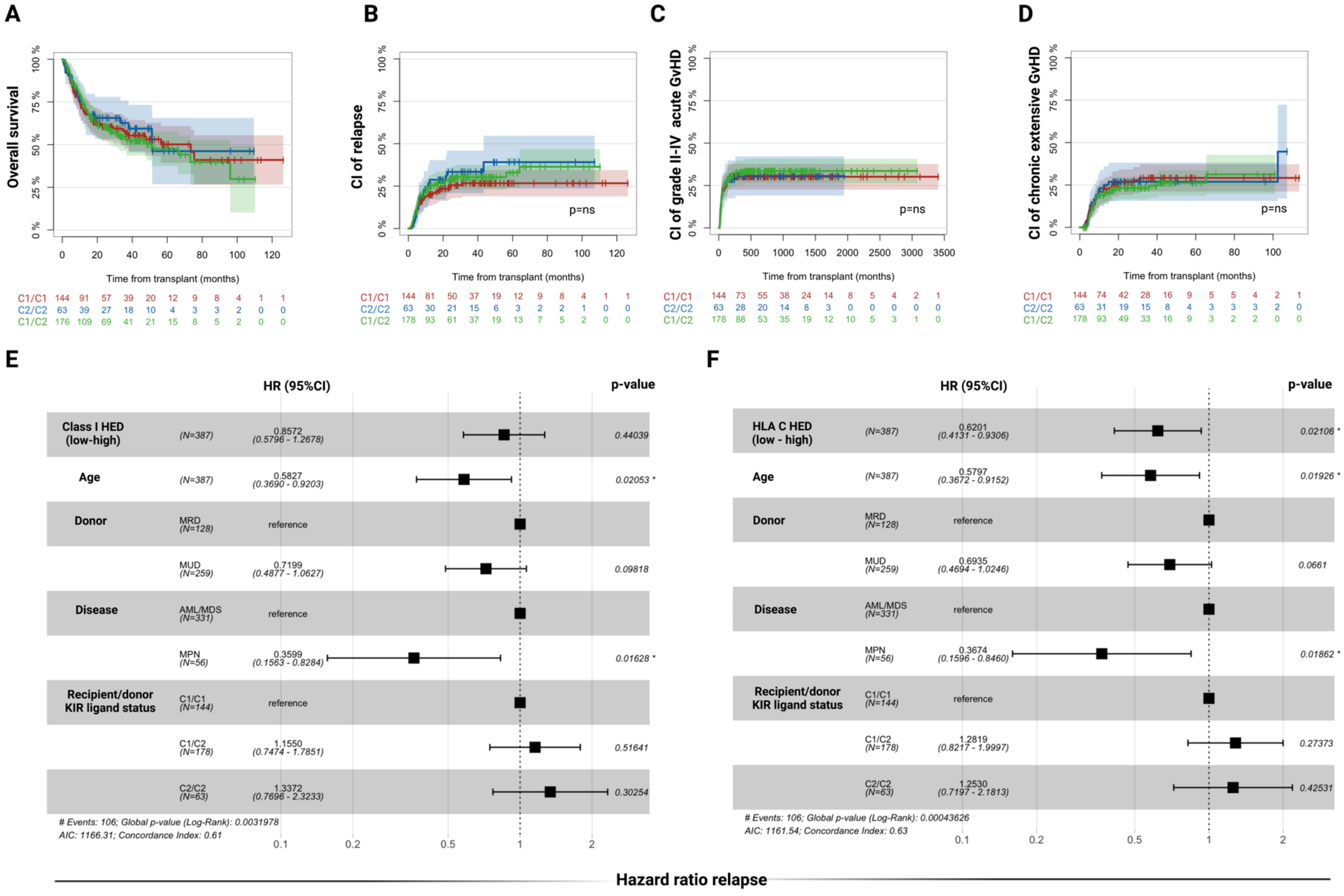

Supplementary Figure 10: Impact of KIR ligand status on HLA-C locus on post-transplant outcomes.
A) Kaplan-Meyer estimates of overall survival according to HLA-C KIR ligand group.
B) Cumulative incidence of relapse according to HLA-C KIR ligand group.
C) Cumulative incidence grade II-IV acute GvHD according to HLA-C KIR ligand group.
D) Cumulative incidence chronic extensive GvHD according to HLA-C KIR ligand group.
Shaded bands represent 95% confident interval.
E) Multivariable cox cause specific models of impact of class I on relapse adjusted for confounding factors, including recipient/donor KIR ligand status.
F) Multivariable cox cause specific models of impact of HLA-C HED on relapse adjusted for confounding factors, including recipient/donor KIR ligand status.
Black squares indicate the odd ratio and error bars the 95% confident intervals.
Non-adjusted p-values indicate the significance of the log-rank test.
Abbreviations: HR: hazard ratio; 95%CI: 95% confident interval; MRD: matched related; MUD: matched unrelated; AML: Acute myeloid leukemias; MDS: myelodysplastic syndromes; MPN: myeloproliferative neoplasms.

Supplementary Figure 11

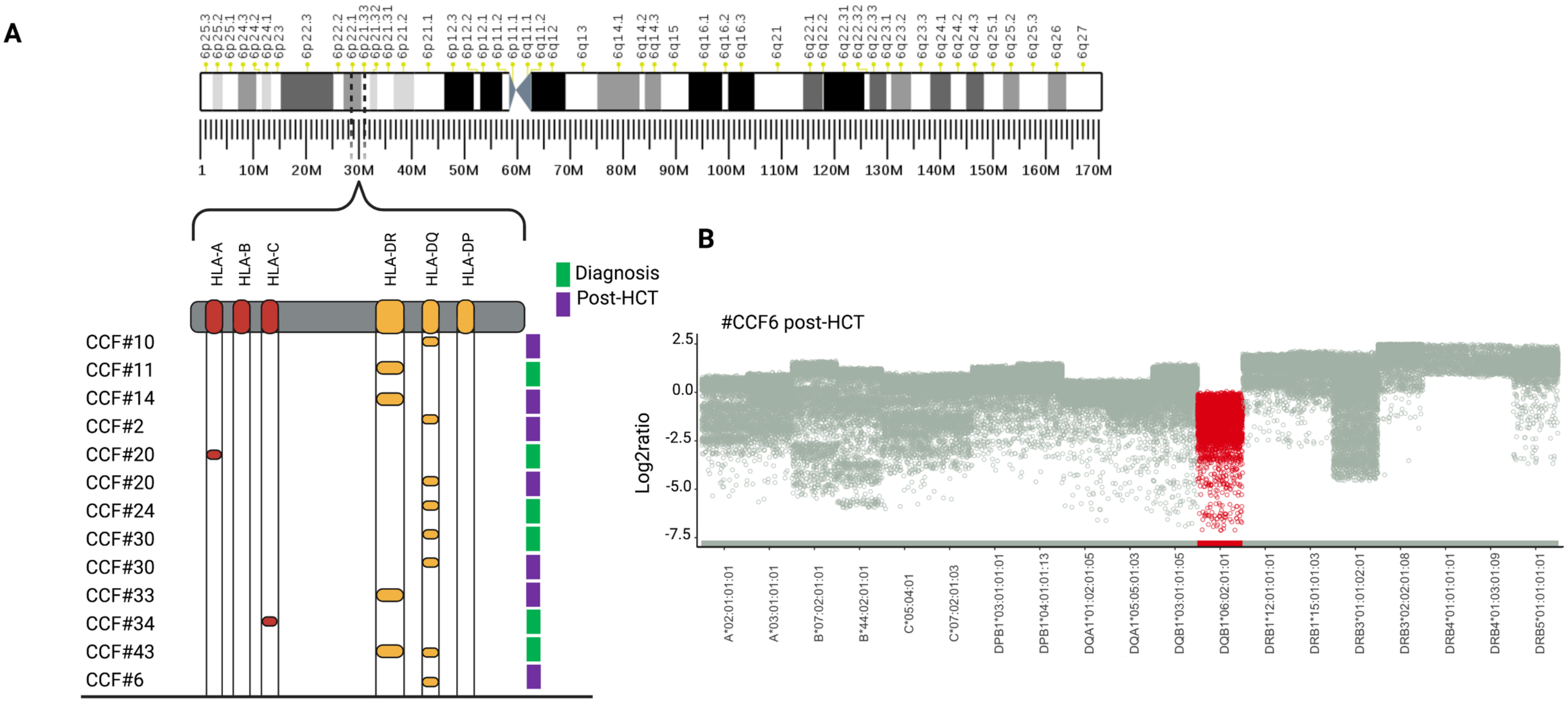

Supplementary Figure 11: Allelic loss events in pre and post-HCT specimens. A) Sketch summarizing the all the somatic allelic losses occurring in 6p region in pre and post-HCT samples. **B)** Scatterplot indicating the Logarithmic base 2 ratio of the base coverage for each allele. Data were retrieved from the HLA sequence information of patient CCF#6 at post-HCT relapse. Each dot indicates a reference base coverage for each allele, and the position of each dot on the y-axis represents the log2 of the base coverage/average locus depth ratio.

REFERENCES

1. Food and Drug Administration, HHS. Human subject protection; foreign clinical studies not conducted under an investigational new drug application. Final rule. *Fed Regist*. 2008;73(82):22800–22816.
2. Angelucci E, Polchi P, Lucarelli G, et al. Allogeneic bone marrow transplantation for hematological malignancies following therapy with high doses of busulphan and cyclophosphamide. *Haematologica*. 1989;74(5):455–461.
3. Fière D, Lepage E, Sebban C, et al. Adult acute lymphoblastic leukemia: a multicentric randomized trial testing bone marrow transplantation as postremission therapy. The French Group on Therapy for Adult Acute Lymphoblastic Leukemia. *J. Clin. Oncol.* 1993;11(10):1990–2001.
4. Eder S, Canaani J, Beohou E, et al. Thiotepa-based conditioning versus total body irradiation as myeloablative conditioning prior to allogeneic stem cell transplantation for acute lymphoblastic leukemia: A matched-pair analysis from the Acute Leukemia Working Party of the European Society for Blood and Marrow Transplantation. *Am. J. Hematol.* 2017;92(10):997–1003.
5. Glucksberg H, Storb R, Fefer A, et al. Clinical manifestations of graft-versus-host disease in human recipients of marrow from HL-A-matched sibling donors. *Transplantation*. 1974;18(4):295–304.
6. Jagasia MH, Greinix HT, Arora M, et al. National Institutes of Health Consensus Development Project on Criteria for Clinical Trials in Chronic Graft-versus-Host Disease: I. The 2014 Diagnosis and Staging Working Group report. *Biol. Blood Marrow Transplant.* 2015;21(3):389-401.e1.
7. Filipovich AH, Weisdorf D, Pavletic S, et al. National Institutes of Health consensus development project on criteria for clinical trials in chronic graft-versus-host disease: I. Diagnosis and staging working group report. *Biol. Blood Marrow Transplant.* 2005;11(12):945–956.
8. Pagliuca S, Gurnari C, Hercus C, et al. Molecular landscape of immune pressure and escape in aplastic anemia. *Leukemia*. 2023;37(1):202–211.
9. Van der Auwera GA, Carneiro MO, Hartl C, et al. From FastQ data to high confidence variant calls: the Genome Analysis Toolkit best practices pipeline. *Curr Protoc Bioinformatics*. 2013;43:11.10.1-11.10.33.
10. Koboldt DC, Zhang Q, Larson DE, et al. VarScan 2: Somatic mutation and copy number alteration discovery in cancer by exome sequencing. *Genome Res.* 2012;22(3):568–576.
11. Robinson J, Barker DJ, Georgiou X, et al. IPD-IMGT/HLA Database. *Nucleic Acids Research*. 2019;gkz950.
12. Gurnari C, Pagliuca S, Kewan T, et al. Is nature truly healing itself? Spontaneous remissions in Paroxysmal Nocturnal Hemoglobinuria. *Blood Cancer J*. 2021;11(11):187.
13. Li H, Durbin R. Fast and accurate short read alignment with Burrows-Wheeler transform. *Bioinformatics*. 2009;25(14):1754–1760.
14. Li H, Handsaker B, Wysoker A, et al. The Sequence Alignment/Map format and SAMtools. *Bioinformatics*. 2009;25(16):2078–2079.
15. Kopanos C, Tsiolkas V, Kouris A, et al. VarSome: the human genomic variant search engine. *Bioinformatics*. 2019;35(11):1978–1980.
