# Supplementary material for: Leukemia relapse via genetic immune escape after allogeneic hematopoietic cell transplantation
Source: Nat Commun. 2023 May 31;14:3153. doi: 10.1038/s41467-023-38113-4 (PMC10232425; doi:10.1038/s41467-023-38113-4)
Supplement: Supplementary file 4 — Description of Additional Supplementary Files [file 41467_2023_38113_MOESM4_ESM.docx]

**Description of Additional Supplementary Files**

Supplementary Data 1

Description: HLA genotypes and HED computation in healthy controls

Supplementary Data 2

Description: Clinical details of patients sequenced for TCR

Supplementary Data 3

Description: Genes investigated from targeted panels for myeloid associated architecture

Supplementary Data 4

Description: Disease characteristics of patients included in RNAseq study

Supplementary Data 5

Description: Immune gene sets studied through whole exome sequencing

Supplementary Data 6

Description: Non-HLA immune related genes impacted by somatic hits

Supplementary Data 7

Description: KIR ligand status and KIR genotype for selected MRD transplants

Supplementary Data 8

Description: Post-HCT TCRvBeta sequencing
